# Stauffer Syndrome as the Initial Presentation of Advanced Metastatic Prostate Cancer

**DOI:** 10.7759/cureus.37663

**Published:** 2023-04-16

**Authors:** Sneha Khanal, Tanushree Bhatt, Irhoboudu Dickson Atogwe, Vikram Itare, Elina Shrestha, Muhammad Sulh

**Affiliations:** 1 Internal Medicine, BronxCare Health System, Bronx, USA; 2 Pathology, BronxCare Health System, Bronx, USA

**Keywords:** clear cell renal cancer, cholestatic jaundice, cholestatic liver disease, paraneoplastic syndromes, prostate cancer (pca), stauffer’s syndrome

## Abstract

Stauffer’s syndrome is a paraneoplastic syndrome that has historically been associated with renal cell carcinoma. It is defined by the anicteric elevation of liver enzymes in the absence of liver metastasis, and the reversibility of clinical and biochemical changes upon treatment of the primary pathology. Here, we discuss the rare presentation of Stauffer’s syndrome in a patient with advanced metastatic prostate cancer. A 72-year-old male presented with generalized weakness, dizziness, weight loss, and icterus who was incidentally found to have a prostatic enlargement on physical examination. The laboratory investigations and radiographic imaging confirmed the diagnosis of metastatic prostatic cancer without any evidence of mechanical biliary obstruction as confirmed by biopsy and imaging. The cancer had metastasized to pelvic sidewalls, pelvic bones, ribs, urinary bladder, and local lymph nodes. Our case signifies that a high index of suspicion for underlying cancer should be maintained in patients presenting with cholestatic liver dysfunction, with or without jaundice, especially in the absence of a recognizable mechanical etiology of cholestasis.

## Introduction

Paraneoplastic hepatic dysfunction in association with renal cell carcinoma had been initially described by Maurice H. Stauffer as Stauffer’s syndrome in 1961. The inceptive diagnostic criteria included anicteric elevation of liver enzymes with an underlying diagnosis of renal cell carcinoma, exclusion of liver metastasis, and reversibility of clinical and biochemical changes upon treatment of the primary pathology ​[1]. However, atypical icteric variants have been described with an incidence of hyperbilirubinemia in 15% of cases ​[2]. Historically, Stauffer’s syndrome was associated with renal cell carcinomas, however, there is increasing literary evidence of other etiologies including prostate, lung, bladder, pancreas, and soft tissue cancers, gastrointestinal carcinoids as well as malignant lymphoproliferative conditions ​[1].​ Here we discuss a rare presentation of a patient with cholestatic jaundice and advanced stage four prostate cancer without evidence of metastasis to the liver as confirmed by liver biopsy and imaging. 

## Case presentation

A 72-year-old male without medical comorbidities presented to the emergency department with complaints of anorexia, weight loss, generalized malaise, weakness, and dizziness worsening over the past two weeks causing multiple falls at home. He also noted yellowing of his sclera, urine, and skin for the same duration. He did not have abdominal pain, vomiting, melena, hematochezia, fever, night sweats, hematuria, frequency, or hesitancy during urination. He denied recent travel and had no surgeries in the past. He was a former heavy drinker (10-20 beers a day since the age of 14) who had been abstinent for eight months. He did not smoke, use any illicit drugs, or take any over-the-counter or herbal medications. The patient did not have any allergies. He had no pets. 

**Figure 1:**
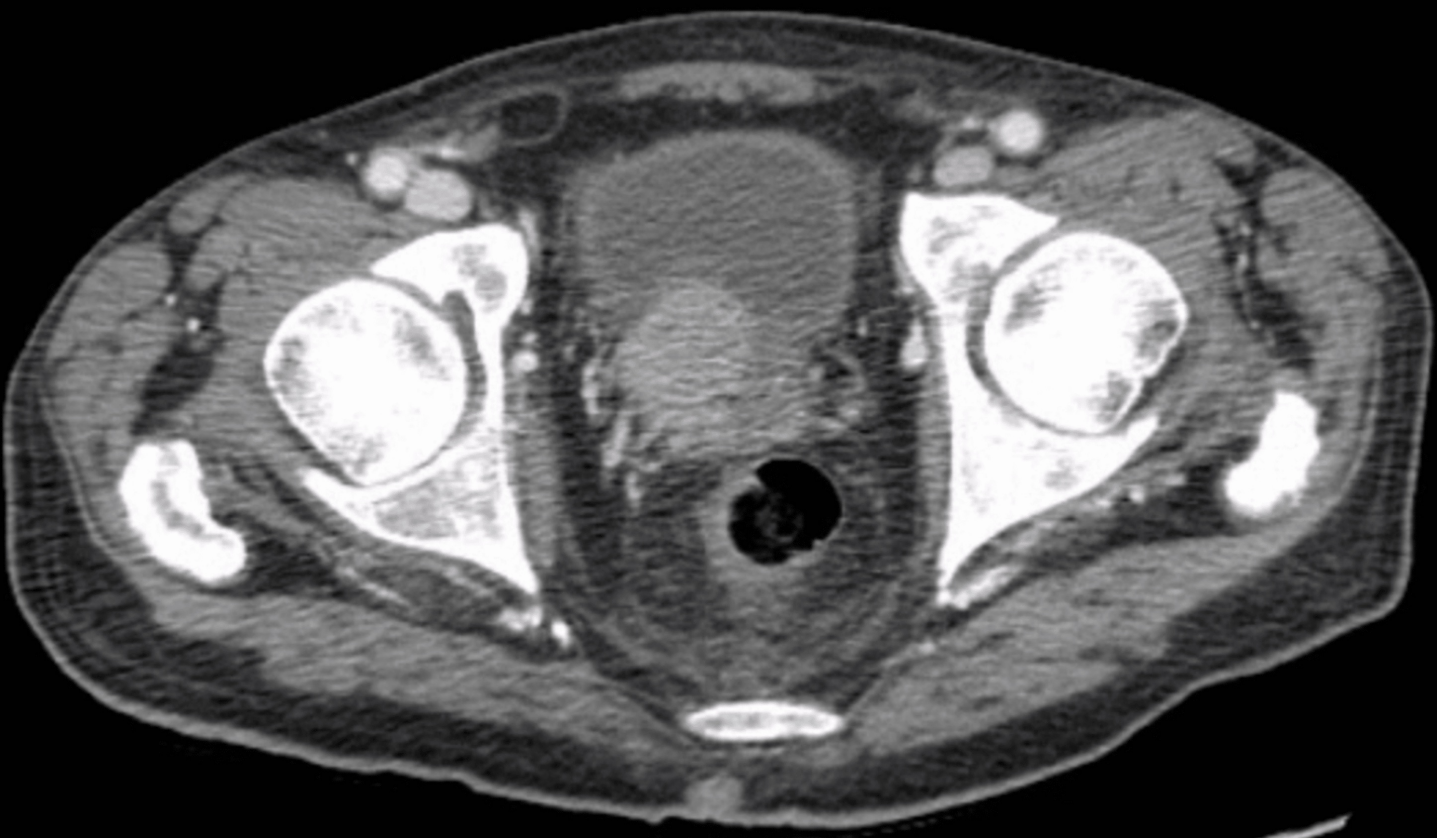
Prostatic mass invading the urinary bladder and local lymph nodes

Upon presentation, the patient was afebrile, normotensive, and had sinus tachycardia. His physical examination revealed icterus, a soft abdomen, and no hepatosplenomegaly. He had a smooth, uniformly enlarged prostate without any mass or tenderness upon rectal examination. Laboratory investigations suggested conjugated hyperbilirubinemia, a cholestatic elevation of liver enzymes, anemia, elevated lipase, and electrolyte derangements including hypokalemia, hypophosphatemia, and hypomagnesemia (Table 1). The patient had elevated gamma-glutamyl transferase (GGT) and a deranged coagulation profile. Prostate-specific antigen was elevated to >5000 ng/ml (reference range: 0.00-4 ng/ml). Infectious and autoimmune hepatitis workups were negative. A Computed Tomography (CT) of the abdomen and pelvis with contrast revealed findings concerning for metastatic prostate cancer (Figure 1).

**Table 1:** Laboratory parameters upon admission

Laboratory parameters	Value	Reference range
Total bilirubin	20.1mg/dl	0.2-1.1 mg/dl
Conjugated bilirubin	18.1 mg/dl	0.0-0.3 mg/dl
Aspartate aminotransferase (AST)	58 U/l	9-48 U/l
Alanine aminotransferase (ALT)	42 U/l	5-40 U/l
Alkaline phosphatase (ALP)	920 U/l	56-155 U/l
Gamma-glutamyl transferase (GGT)	963 U/l	8-54 U/l
Albumin	4.0 g/dl	3.2-4.6 g/dl
Hemoglobin	10.5 gm/dl	12-16 gm/dl
Hematocrit	30.5%	42-51%
White blood cells	6.1 K/ul	4.8-10.8 K/ul
Platelets	199 K/ul	150-400 K/ul
Lipase	112 U/l	<61 U/l
Prothrombin time (PT)	16.9 seconds	9.9-13.3 seconds
Activated partial thromboplastin time (aPTT)	30.2 seconds	27.2-39.6 seconds
International normalized ratio (INR)	1.42	0.85-1.14

**Figure 2:**
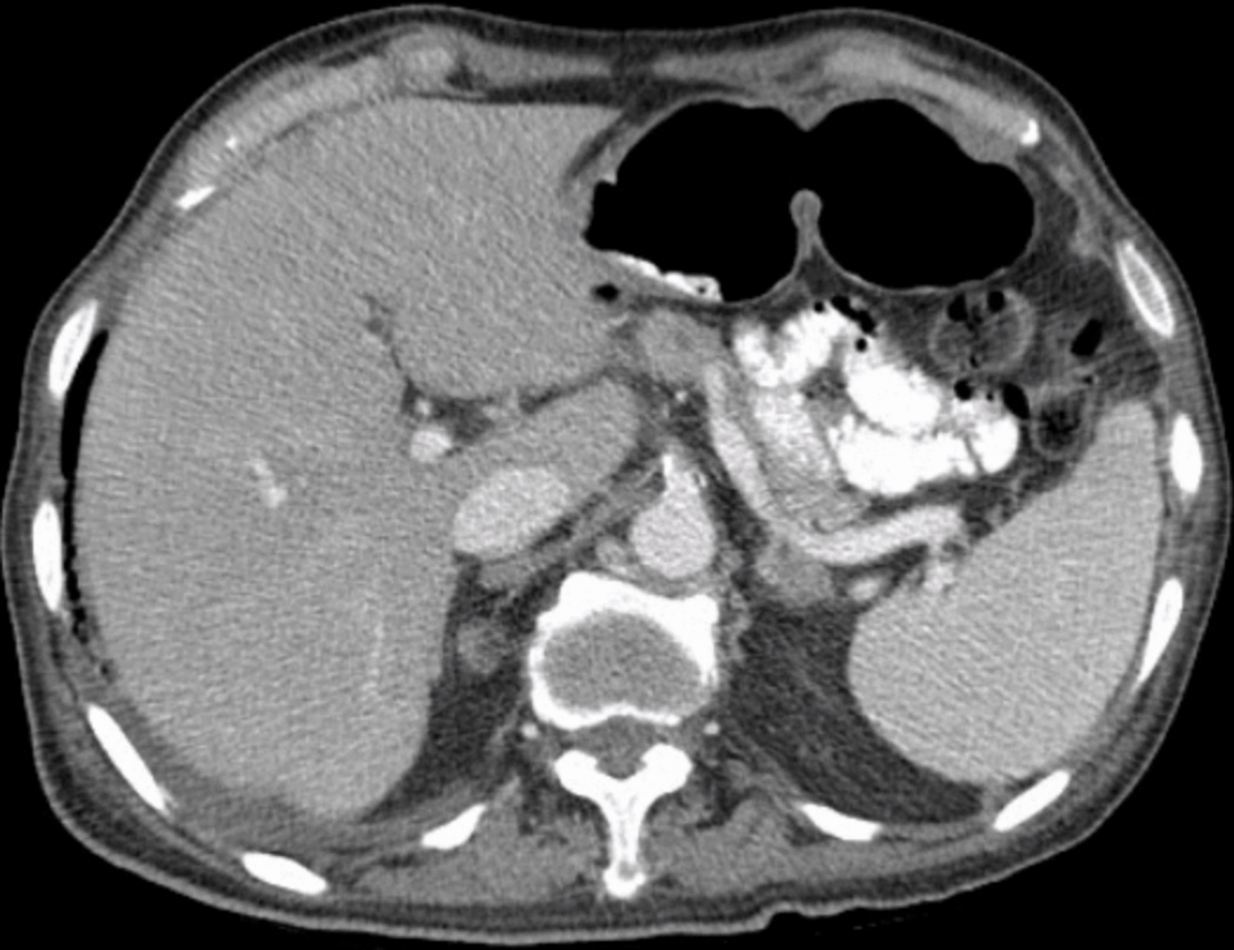
No aggressive focal hepatic lesions noted in the CT abdomen and pelvis

Metastases to bilateral pelvic sidewalls, pelvic bones, and multiple ribs were noted and the prostatic mass had invaded the urinary bladder and local lymph nodes. Hepatic steatosis was noted without any aggressive focal hepatic lesions (Figure 2) along with an incidental left adrenal adenoma.

**Figure 3:**
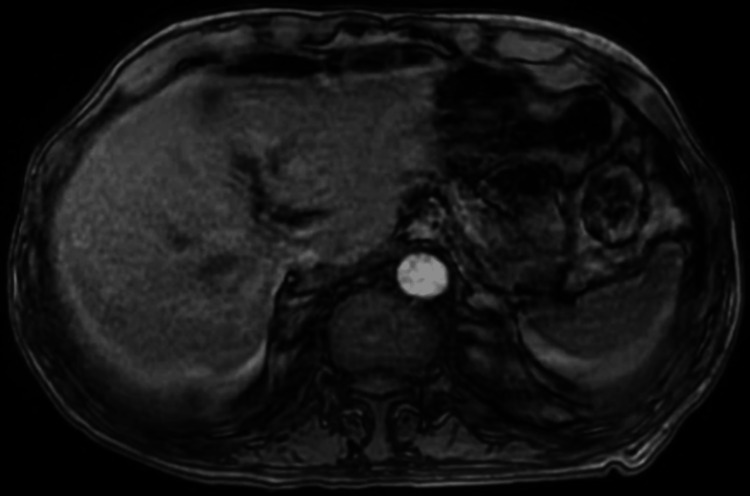
MRI of the abdomen showing no evidence of intrahepatic or common duct dilation, abnormal enhancement, mass, or extrinsic mass effect.

**Figure 4:**
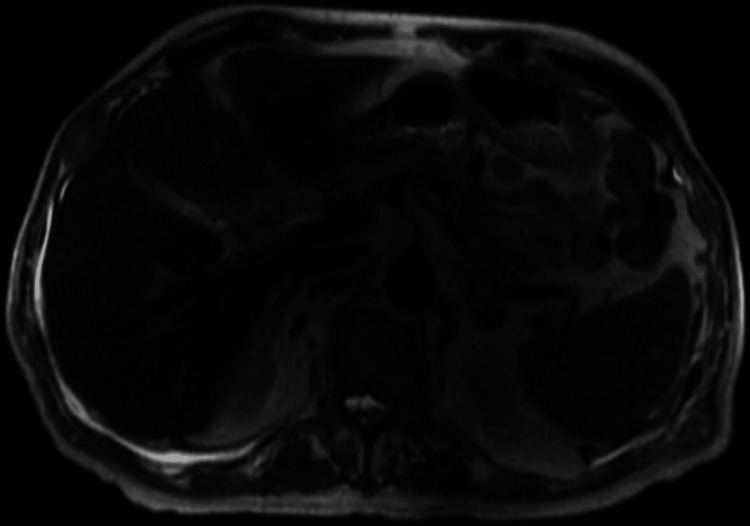
Magnetic resonance cholangiopancreatography (MRCP) of the abdomen showing no evidence of intrahepatic or common duct dilation, abnormal enhancement, mass, or extrinsic mass effect.

Ultrasound of the abdomen showed heterogeneous liver without suspicious lesions. Due to the concern for the presence of urokinase-plasminogen activator in prostate cancer that activates fibrinolysis upon prostate biopsy, the patient was planned to undergo a bone biopsy instead for tissue diagnosis. Magnetic resonance imaging (MRI) and magnetic resonance cholangiopancreatography (MRCP) of the abdomen showed no evidence of intrahepatic or common bile duct dilation, abnormal enhancement, mass, or extrinsic mass effect on the biliary or pancreatic ducts (Figures 3, 4).

**Figure 5:**
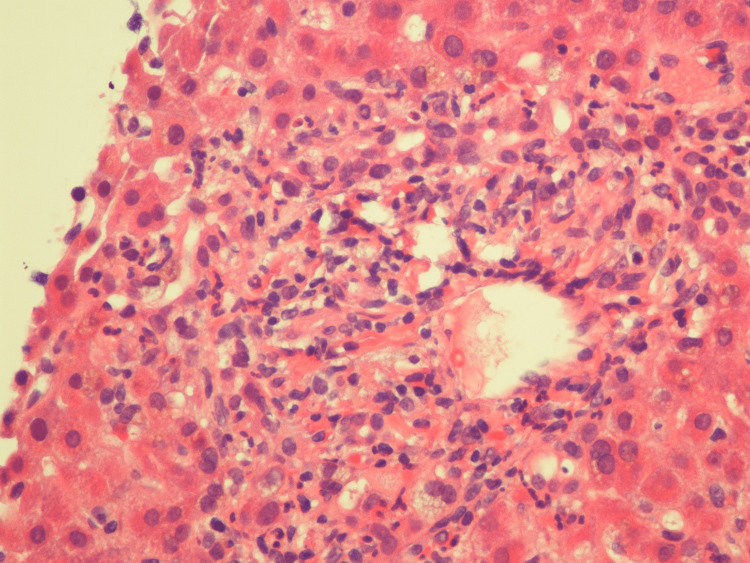
Liver parenchyma with foci of bile stasis, canalicular pattern with acute inflammation of the bile duct in the portal triad

**Figure 6:**
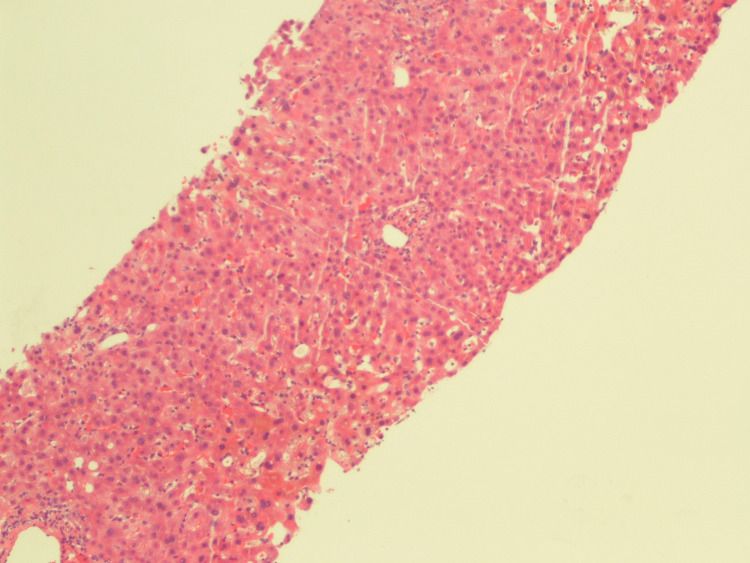
Liver parenchyma with foci of bile stasis, canalicular pattern with acute inflammation of the bile duct in the portal triad, and no evidence of granulomatous inflammation or malignancy.

A liver biopsy was planned to evaluate the cause of persistent conjugated hyperbilirubinemia. A CT-guided liver biopsy revealed liver parenchyma with foci of bile stasis, canalicular pattern with acute inflammation of the bile duct in the portal triad, and no evidence of granulomatous inflammation or malignancy (Figures 5, 6). Unfortunately, the patient had a sudden cardiac arrest and passed away despite all resuscitative efforts. 

## Discussion

Cholestatic jaundice can be a manifestation of multiple conditions, including dynamic or adynamic bile duct obstruction, acute and chronic viral hepatitis, or autoimmune disorders like primary biliary cirrhosis and primary sclerosing cholangitis ​[3]. Cholestasis in the presence of malignancy is usually secondary to metastases and mechanical obstruction ​[4]. However, the absence of obstruction and other etiologies with underlying prostate cancer describes the paraneoplastic nature of Stauffer’s syndrome. The pathophysiology of Stauffer’s syndrome is unclear; nevertheless, it has been hypothesized that increased circulating interleukin-6 (IL-6) could contribute to its immunopathogenesis ​[5]. Prior studies have demonstrated that the administration of anti-IL-6 monoclonal antibodies reversed most biochemical derangements in patients with renal cell cancer ​[6]. 

Stauffer’s syndrome is characterized by a cholestatic pattern of liver enzyme elevation, with or without jaundice, hypergammaglobulinemia, elevated alkaline phosphatase, hypoalbuminemia, prolonged prothrombin time, elevated erythrocyte sedimentation rate, elevated alpha-2-globulin, and hepatosplenomegaly ​[7]​. The temporality and reversibility of this condition in association with the treatment of malignancy further support the diagnosis ​[8]​. Biopsy and histopathological analysis of the liver vary from normal liver histology to fibrotic changes or degeneration of hepatocytes ​[9]​. 

Early-stage prostate cancer is often asymptomatic. Paraneoplastic syndromes, such as Stauffer syndrome, may be early indicators of disease; thus a high index of suspicion for underlying malignancy should be maintained in patients with cholestatic liver dysfunction in the absence of common diagnostic findings of more common etiologies. A rapid down-spiraling clinical course can ensue from metastatic prostate cancer leading to rapid decompensation and death despite supportive measures ​[10]​. Hence, a high index of suspicion for underlying cancer should always be maintained in patients presenting with cholestatic liver dysfunction, with or without jaundice, especially in the absence of a recognizable etiology of cholestasis. 

## Conclusions

While renal cell cancer is the primary cause of Stauffer's syndrome, prostate cancer ranks second in prevalence. However, it is crucial to look for other potential malignancies that could contribute to the syndrome. Detecting this paraneoplastic condition at an early stage can result in the complete reversal of symptoms and better treatment outcomes. There is a possibility of developing targeted treatments for managing Stauffer's syndrome by conducting further research on its pathophysiology.
