# Correction: Stauffer Syndrome as the Initial Presentation of Advanced Metastatic Prostate Cancer

**DOI:** 10.7759/cureus.c194

**Published:** 2024-09-30

**Authors:** Sneha Khanal, Tanushree Bhatt, Irhoboudu Dickson Atogwe, Vikram Itare, Elina Shrestha, Muhammad Sulh

**Affiliations:** 1 Internal Medicine, BronxCare Health System, Bronx, USA; 2 Pathology, BronxCare Health System, Bronx, USA

This article has been corrected to address the following error: in the first sentence of the Introduction, the authors incorrectly state the name of Dr. Maurice H. Stauffer as "Herbert Maurice Stauffer". This has now been fixed.

